# Supplementary material for: Tumor-Extrinsic Axl Expression Shapes an Inflammatory Microenvironment Independent of Tumor Cell Promoting Axl Signaling in Hepatocellular Carcinoma
Source: Int J Mol Sci. 2024 Apr 10;25(8):4202. doi: 10.3390/ijms25084202 (PMC11050718; doi:10.3390/ijms25084202)
Supplement: Supplementary file 1 [file ijms-25-04202-s001.zip › Supplementary Material and Methods_Breitenecker et al.pdf]

Supplementary Material and Methods

1.1. Sample preparation for mass spectrometry analysis

Cell pellets were resuspended in lysis buffer (8 M urea (VWR, 0568-500G), 50 mM Tris-HCl pH8.0, 150 mM NaCl, 1 mM PMSF, 5 mM sodium butyrate (Sigma-Aldrich, 303410), 10 ng/ml NAM (Sigma Aldrich, 72340), 10 ng/ml trichostatin A (TSA) (Sigma Aldrich, T8552-1MG), 1x cOmplete protease inhibitor cocktail (+EDTA) (Roche, 11697498001), 250 U/replicate benzonase (Merck, 1.01695.0001) and lysed using a Bioruptor sonication device (Diagenode) (settings: 30 sec sonication (power level H, 30 sec cooling, 5 cycles). Lysates were centrifuged at 15,000 x g for 10 min at 4°C and precipitated with 4x volumes of cold (-20°C) 100% acetone overnight at 4°C.

Protein pellets were washed with cold (-20°C) 80% acetone, air dried for 5 min, dissolved in 8 M urea, 50 mM ABC (Sigma Aldrich, 09830-500G), reduced with 10 mM DTT (Roche, 10197777001) for 45 min at room temperature, and subsequently carbamidomethylated with 20 mM IAA (Sigma Aldrich, I6125-5G) for 30 min at room temperature in the dark. The alkylation reaction was quenched by adding 5 mM DTT for 10 min. The urea concentration was reduced to 4 M using 50 mM ABC. Samples were pre-digested for 90 min at 37°C with Lys-C (FUJIFILM Wako Pure Chemical Corporation, 125-02543) at an enzyme-to-substrate ratio of 1:100 and subsequently reduced to 1 M Urea using 50 mM ABC and digested overnight at 37°C with sequencing grade trypsin (Trypsin Gold, Mass Spec Grade, Promega, V5280) at an enzyme-to-substrate ratio of 1:50. Digests were stopped by acidification with TFA (Thermo Scientific, 28903; 0.5% final concentration) and desalted on a 50 mg tC18 Sep-Pak cartridge (Waters, WAT054960). Peptide concentrations were determined and adjusted according to UV chromatogram peaks obtained with an UltiMate 3000 Dual LC nano-HPLC System (Dionex, Thermo Fisher Scientific; Waltham; USA), equipped with a monolithic C18 column (Phenomenex; Torrance; USA). Desalted samples were concentrated in a SpeedVac concentrator (Eppendorf; Hamburg; Germany) and subsequently lyophilized overnight.

Lyophilized peptides were dissolved in 50 µl 100 mM TEAB (Sigma; St. Louis; USA). 450 µg of each TMT pro reagent (Thermo Fisher Scientific; Waltham; USA) were dissolved in 30 µl of 100% anhydrous acetonitrile and 30 µl was added to the 20µl/300µg peptide/TEAB mixes. Labels used: 126C: MR 1; 127C: MR 2; 127N: MR-Axl 1; 128N: MR-Axl 2; 129C: MR 3; 129N: MR-Axl 3; 130C: MR 1+Gas6; 130N: MR-Axl 1+Gas6; 131C MR-Axl 2+Gas6; 131N: MR-Axl +Gas6; 132C: MR3+Gas6; 132N: MR-Axl 3+Gas6. Samples were labeled for 60 min at room temperature. 0.1 µl of each sample were pooled, mixed with 10 µl 0.1% TFA, and analyzed by mass spectrometry (MS) to check labeling efficiency. For quenching, 8 µl of 5% hydroxylamine was added and the reaction was incubated for 25 min at room temperature. Samples were pooled and subsequently desalted with Sep-Pak tC-18 cartridges (Waters). Desalted samples were dried for 30 min in a SpeedVac vacuum centrifuge and subsequently lyophilized overnight.

10% TFA was used to adjust the pH of the mix to less than 7 before neutral pH fractionation was performed using a 60 min gradient of 4.5 to 45% ACN (VWR, 83639.320) in 10 mM ammonium formate (1 ml formic acid, Merck, 1.11670.1000), 3ml ammonia (13N) (1.05432.1000) in 300 ml H_2O, pH = 7 - 8, dilute 1:10) on an UltiMate 3000 Dual LC nano-HPLC System (Dionex, Thermo Fisher Scientific; Waltham; USA) equipped with a XBridge Peptide BEH C18 (130 Å, 3.5 µm, 4.6 mm x 250 mm) column (Waters) (flow rate of 1.0 ml / min). Fractions were collected and subsequently pooled in a non-contiguous manner into 8 pools, and lyophilized overnight.

Phosphorylated peptides were enriched using TiO2. An aliquot (peptide: TiO2 resin = 1:6) of TiO2 (Titansphere TiO, GL Sciences, 5020-75000) was washed twice with 50% methanol (Fisher chemical, A456-212) and twice with glycolic acid solution (1 M glycolic acid (Sigma Aldrich, 124737-25G), 70% ACN (VWR, 83639.320), 3% TFA (Thermo Scientific, 28903; Waltham; USA). Lyophilized peptides were dissolved in glycolic acid solution, mixed with the TiO2 resin, and incubated rotating at room temperature for 30 min. Samples were loaded onto Mobicol columns (MoBiTec, M1003, M2110) and shortly centrifuged in a table centrifuge to remove unphosphorylated peptides. Phosphorylated peptides will bind to the TiO2 resin. The resin was washed twice with glycolic acid solution, twice with 200 µl 70%, ACN 3%, TFA and twice with 1% ACN, 0,1% TFA. Phosphorylated peptides were eluted twice using 150 µl 300 mM ammonium hydroxide (VWR, 1.05432.1000). Eluates were united and immediately acidified with conc. TFA to a pH of 2,5. Samples were desalted using a standard C18 (Empore, 2215-C18) StageTip protocol, dried using a SpeedVac (Eppendorf; Hamburg; Germany) and dissolved in 20 ml 2% ACN, 0,1% TFA.

1.2. Liquid chromatography mass spectrometry analysis

The analysis was performed on an UltiMate 3000 Dual LC nano-HPLC System (Dionex, Thermo Fisher Scientific), containing both a trapping column for peptide concentration (PepMap C18, 5x 0.3 mm, 5 µm particle size) and an analytical column (PepMap C18, 500 x 0.075 µm, 2 µm particle size, Thermo Fisher Scientific), coupled to an Orbitrap Exploris 480 mass spectrometer (Thermo Fisher) via a FAIMS Pro ion source (Thermo Fisher). The instruments were operated in data-dependent acquisition (DDA) mode with dynamic exclusion enabled. For peptide separation on the HPLC, the concentration of organic solvent (acetonitrile, VWR, 83639.320) was increased from 2% to 40% in 0.1% formic acid at a flow rate of 230

nl/min, using a 3 hrs gradient time for proteome analysis. Peptides were ionized with a spray voltage of 2,4 kV. MS1 survey scans were recorded with the following parameters: resolution 60,000, scan range 350-1,500 m/z, automatic gain control (AGC) target = Custom, and maximum injection time (IT) mode = Auto, FAIMS voltages on, FAIMS CV -40V, -55V and -70V, dynamic exclusion was set to 45 sec. MS2 analysis was performed with the following parameters: resolution 30,000, normalized AGC Target 150%, AGC target = Custom, maximum IT mode = Custom, isolation window 0.7 m/z, and HCD normalized collision energy of 34%. Total cycle time for each CV was set to 2 sec.
