# Supplementary material for: Tumor-Extrinsic Axl Expression Shapes an Inflammatory Microenvironment Independent of Tumor Cell Promoting Axl Signaling in Hepatocellular Carcinoma
Source: Int J Mol Sci. 2024 Apr 10;25(8):4202. doi: 10.3390/ijms25084202 (PMC11050718; doi:10.3390/ijms25084202)
Supplement: Supplementary file 1 [file ijms-25-04202-s001.zip › Supplementary Table_Breitenecker et al.pdf]

Supplementary Table

Table S1: Quantitative PCR primers.

**Target**	**Sequence forward (5′-3′)**	**Sequence reverse (5′-3′)**
human *28S*	CAGTTCTCTTGGGAATCCAG	TTCAGCAAAGGAGTCAATCCAC
human *CDH1*	ACCACCTCCACAGCCACCGT	GCCCACGCCAAAGTCCTCGG
human *VIMENTIN*	ATTCCACTTTGCGTTCAAGG	CTTCAGAGAGAGGAAGCCGA
human *SNAI1*	GTAATGGCTGTCACTTGTCG	TGTAAACATCTTCCTCCCAGG
human *SNAI2*	TGACCTGTCTGCAAATGCTC	CAGACCCTGGTTGCTTCAA
human *CDH2*	CCACCTTAAAATCTGCAGGC	GTGCATGAAGGACAGCCTCT
mouse 28S	ATACCGGCACGAGACCGATAGTCA	GCGGACCCCACCCGTTTACCTC
mouse *Cdh1*	CAGGTCTCCTCATGGCTTTGC	CAGGTCTCCTCATGGCTTTGC
mouse *Vimentin*	CGGCTGCGAGAGAAATTGC	CCACTTTTCCGTTCAAGGTCAAG
mouse *Snai1*	CACACGCTGCCTTGTGTCT	GGTCAGCAAAAGCACGGTT
mouse *Snai2*	GCGAACTGGACACACACACAGTTAT	GCTGCCGACGATGTCCATACAGTAAT
mouse *Pd-l1*	AACGCCACAGCGAATGATGT	ACAGGATGGATCCCAGAAGC
mouse *Ccl9*	CCCTCTCCTTCCTCATTCTTACA	AGTCTTGAAAGCCCATGTGAAA
mouse *Cxcl2*	CCAACCACCAGGCTACAG	GCGTCACACTCAAGCTCTG
mouse *Ccl2*	AAGCCAGCTCTCTCTTCCTCCA	GCGTTAACTGCATCTGGCTGA
mouse *Cxcl4*	GTTCCCCAGCTCATAGCCACC	TTATATAGGGGTGCTTGCCGGT
mouse *P-Selectin*	CCTCCCGAATGTCAAGCTGT	TCATCGCACATGAACTGGCA
mouse *Vegfa*	GGACGTCTACCAGCGAAGCT	CACAGGACGGCTTGAAGATGT
mouse *Cxcl5*	TGCCCTACGGTGGAAGTCAT	AGCTTTCTTTTTGTCACTGCCC
mouse *Vcam1*	TCTTGGGAGCCTCAACGGTA	CAAGTGAGGGCCATGGAGTC
