# Supplementary material for: Tumor-Extrinsic Axl Expression Shapes an Inflammatory Microenvironment Independent of Tumor Cell Promoting Axl Signaling in Hepatocellular Carcinoma
Source: Int J Mol Sci. 2024 Apr 10;25(8):4202. doi: 10.3390/ijms25084202 (PMC11050718; doi:10.3390/ijms25084202)
Supplement: Supplementary file 1 [file ijms-25-04202-s001.zip › Supplementary Figures_Breitenecker et al.pptx]

## Slide 1
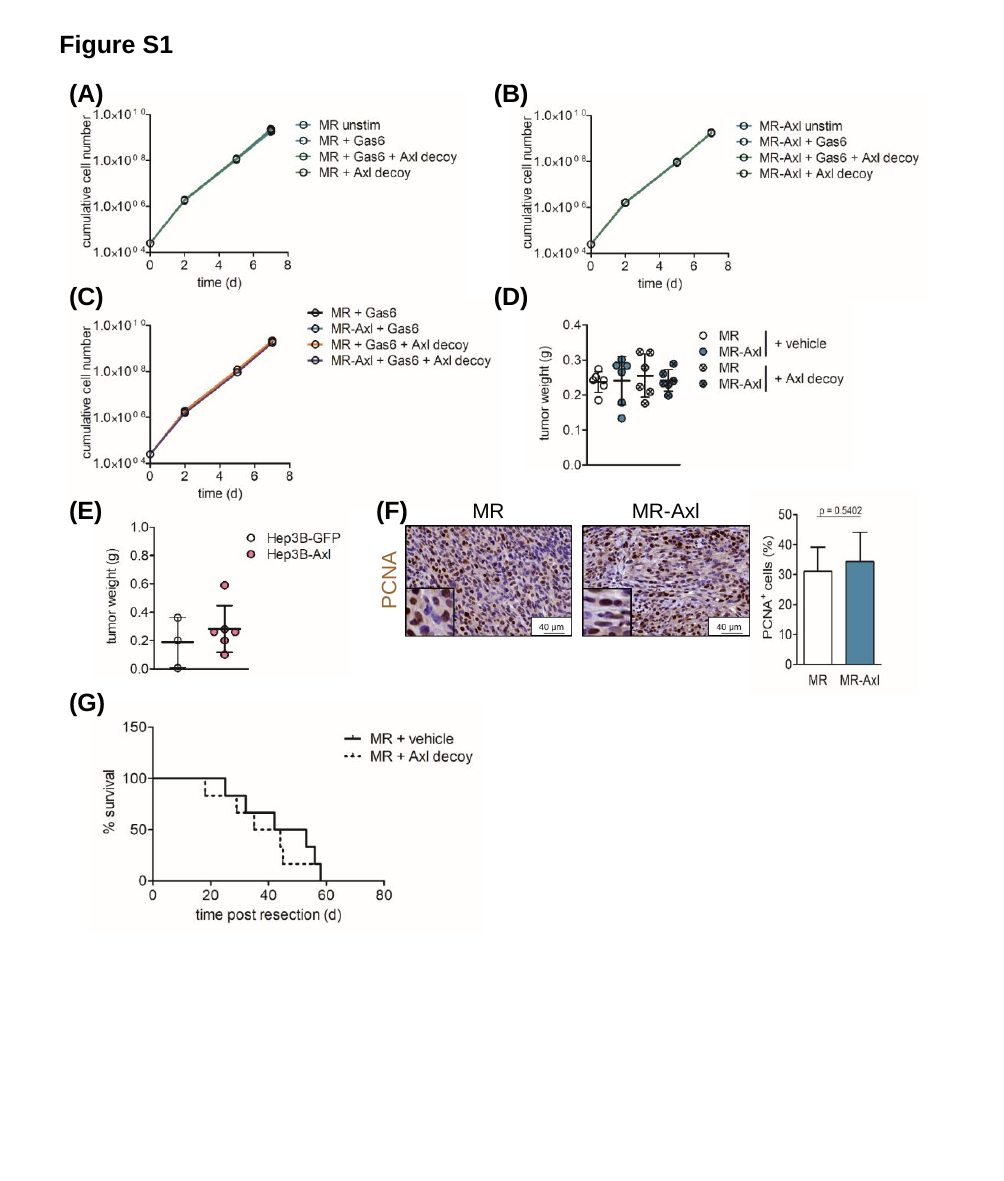

Figure S1
(A)
(B)
(C)
(D)
(E)
(F)
MR
MR-Axl
PCNA
(G)

## Slide 2
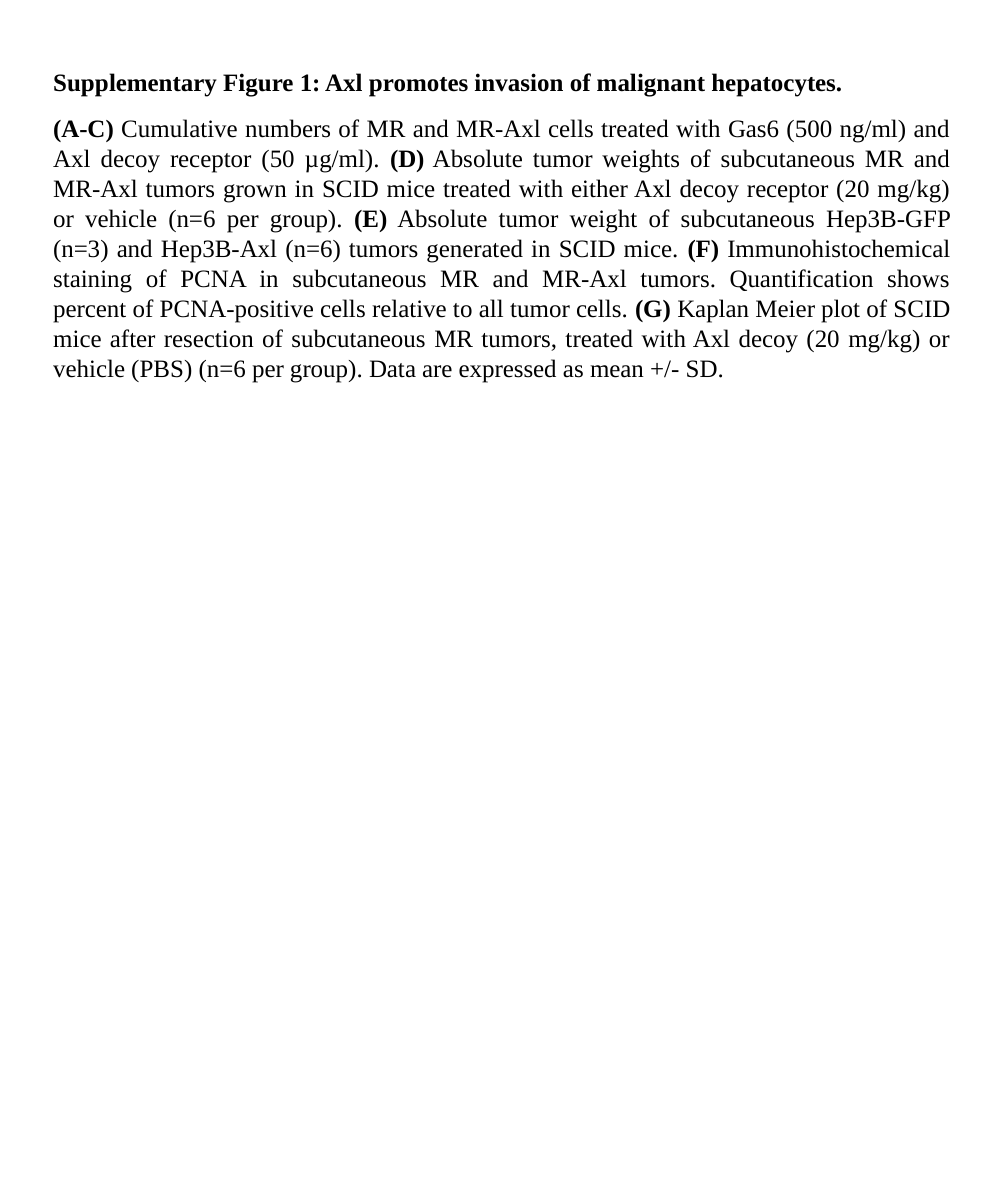

Supplementary Figure 1: Axl promotes invasion of malignant hepatocytes.
(A-C) Cumulative numbers of MR and MR-Axl cells treated with Gas6 (500 ng/ml) and Axl decoy receptor (50 µg/ml). (D) Absolute tumor weights of subcutaneous MR and MR-Axl tumors grown in SCID mice treated with either Axl decoy receptor (20 mg/kg) or vehicle (n=6 per group). (E) Absolute tumor weight of subcutaneous Hep3B-GFP (n=3) and Hep3B-Axl (n=6) tumors generated in SCID mice. (F) Immunohistochemical staining of PCNA in subcutaneous MR and MR-Axl tumors. Quantification shows percent of PCNA-positive cells relative to all tumor cells. (G) Kaplan Meier plot of SCID mice after resection of subcutaneous MR tumors, treated with Axl decoy (20 mg/kg) or vehicle (PBS) (n=6 per group). Data are expressed as mean +/- SD.

## Slide 3
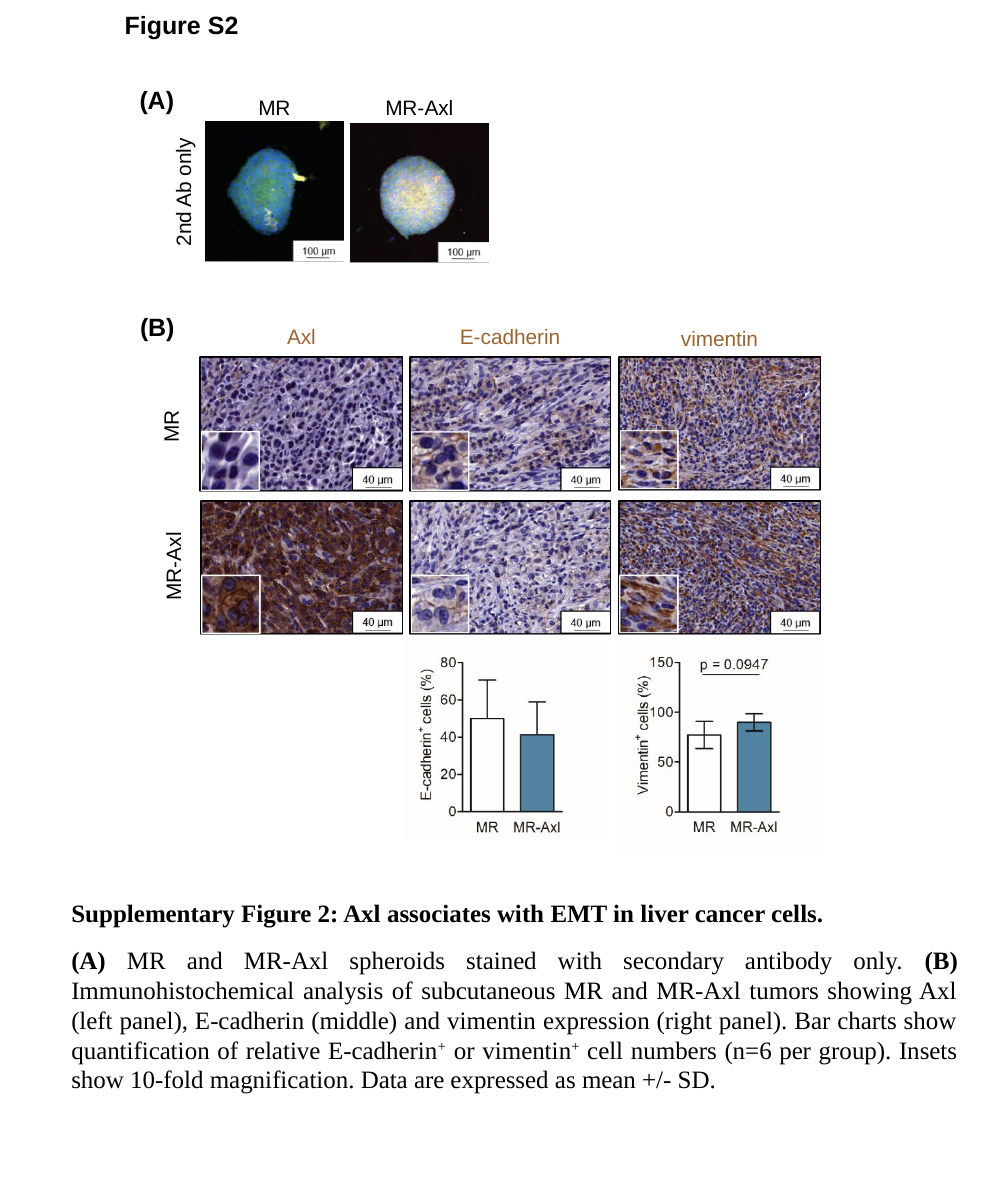

Figure S2
(A)
MR
MR-Axl
2nd Ab only
(B)
Axl
E-cadherin
vimentin
MR
MR-Axl
Supplementary Figure 2: Axl associates with EMT in liver cancer cells.
(A) MR and MR-Axl spheroids stained with secondary antibody only. (B) Immunohistochemical analysis of subcutaneous MR and MR-Axl tumors showing Axl (left panel), E-cadherin (middle) and vimentin expression (right panel). Bar charts show quantification of relative E-cadherin+ or vimentin+ cell numbers (n=6 per group). Insets show 10-fold magnification. Data are expressed as mean +/- SD.

## Slide 4
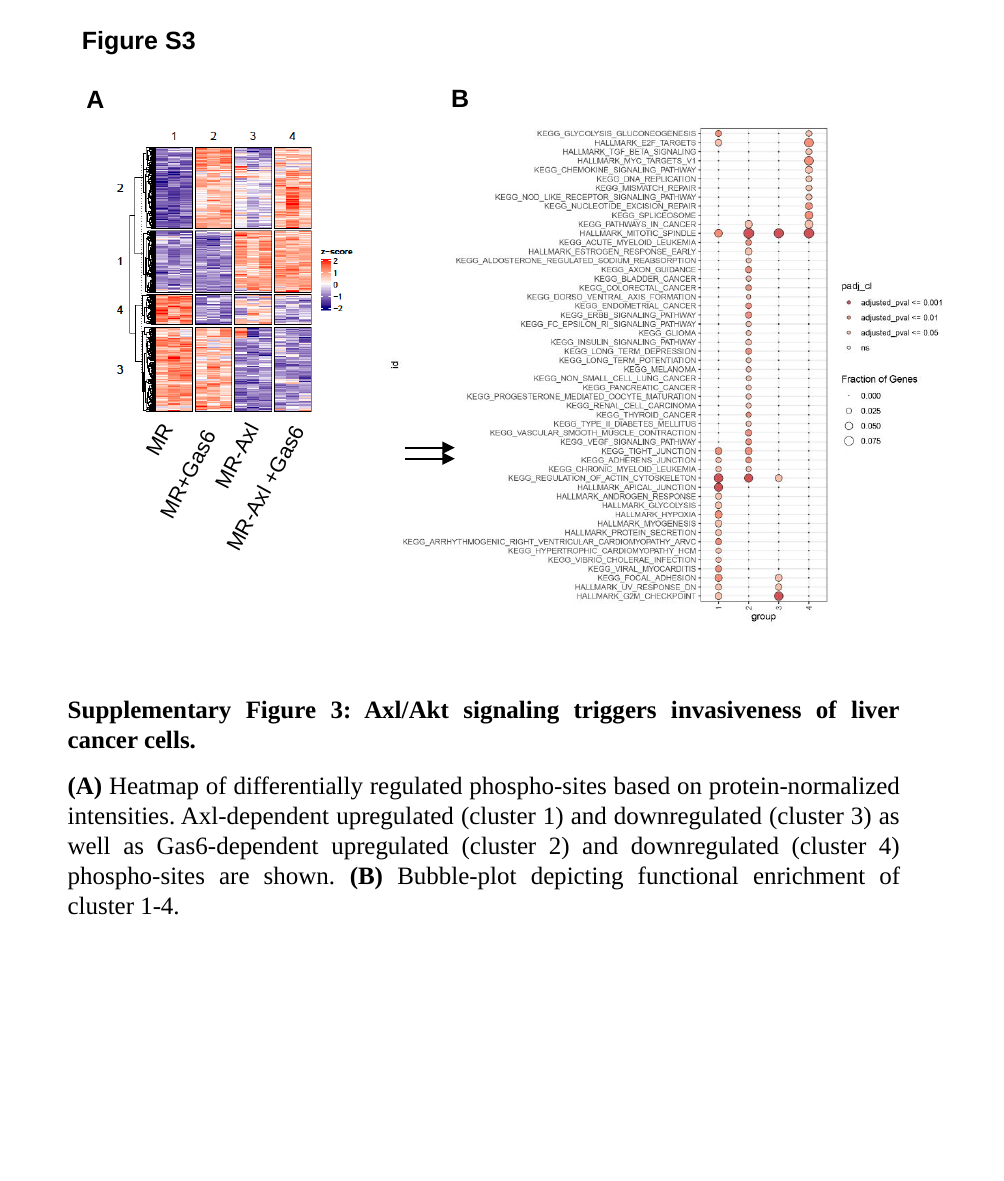

Figure S3
B
A
MR
MR-Axl
MR+Gas6
MR-Axl +Gas6
Supplementary Figure 3: Axl/Akt signaling triggers invasiveness of liver cancer cells.
(A) Heatmap of differentially regulated phospho-sites based on protein-normalized intensities. Axl-dependent upregulated (cluster 1) and downregulated (cluster 3) as well as Gas6-dependent upregulated (cluster 2) and downregulated (cluster 4) phospho-sites are shown. (B) Bubble-plot depicting functional enrichment of cluster 1-4.

## Slide 5
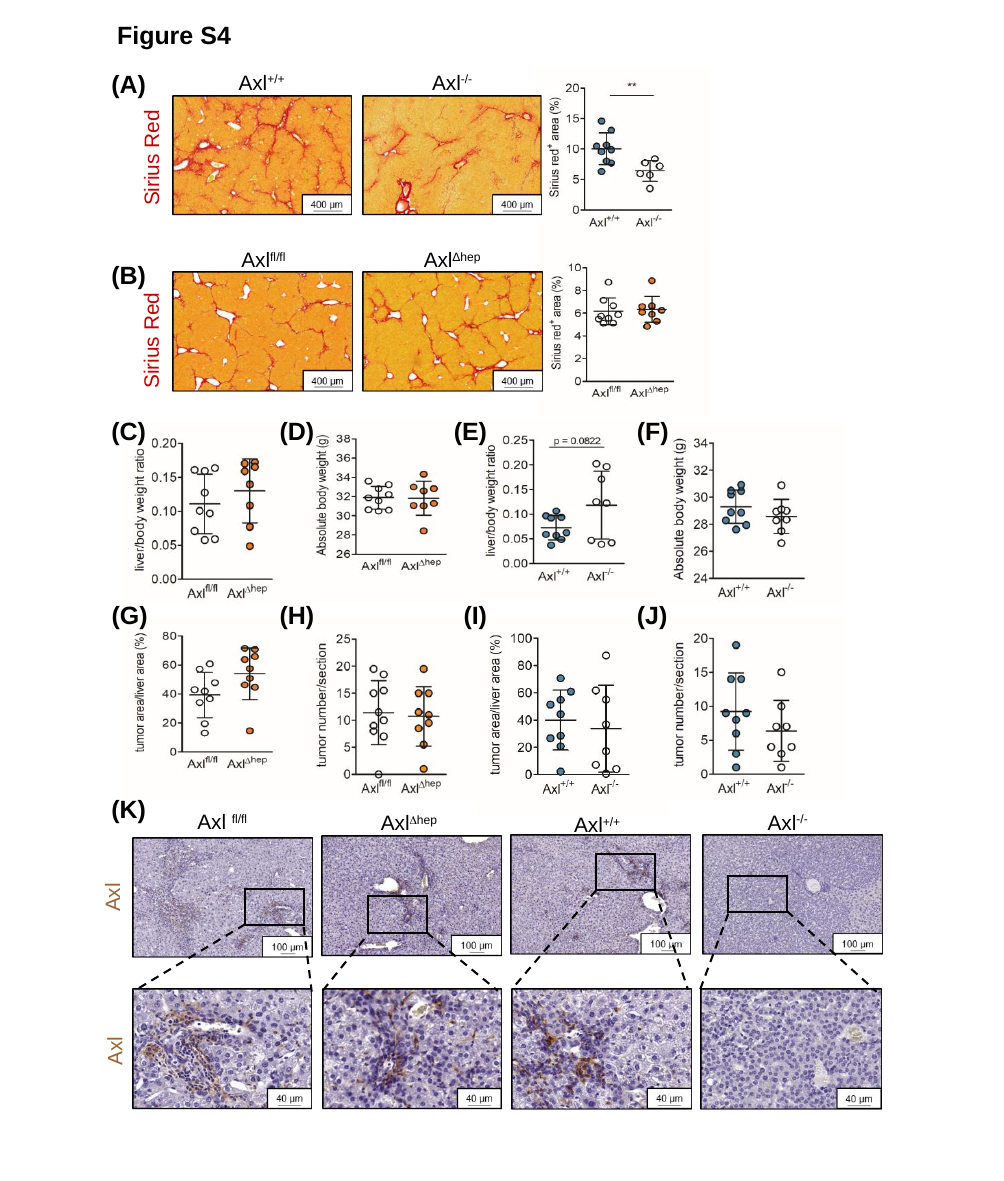

Figure S4
(A)
Axl+/+
Axl-/-
Sirius Red
Axlfl/fl
AxlΔhep
(B)
Sirius Red
(C)
(D)
(E)
(F)
(G)
(H)
(I)
(J)
(K)
Axl fl/fl
Axl-/-
AxlDhep
Axl+/+
Axl
Axl

## Slide 6
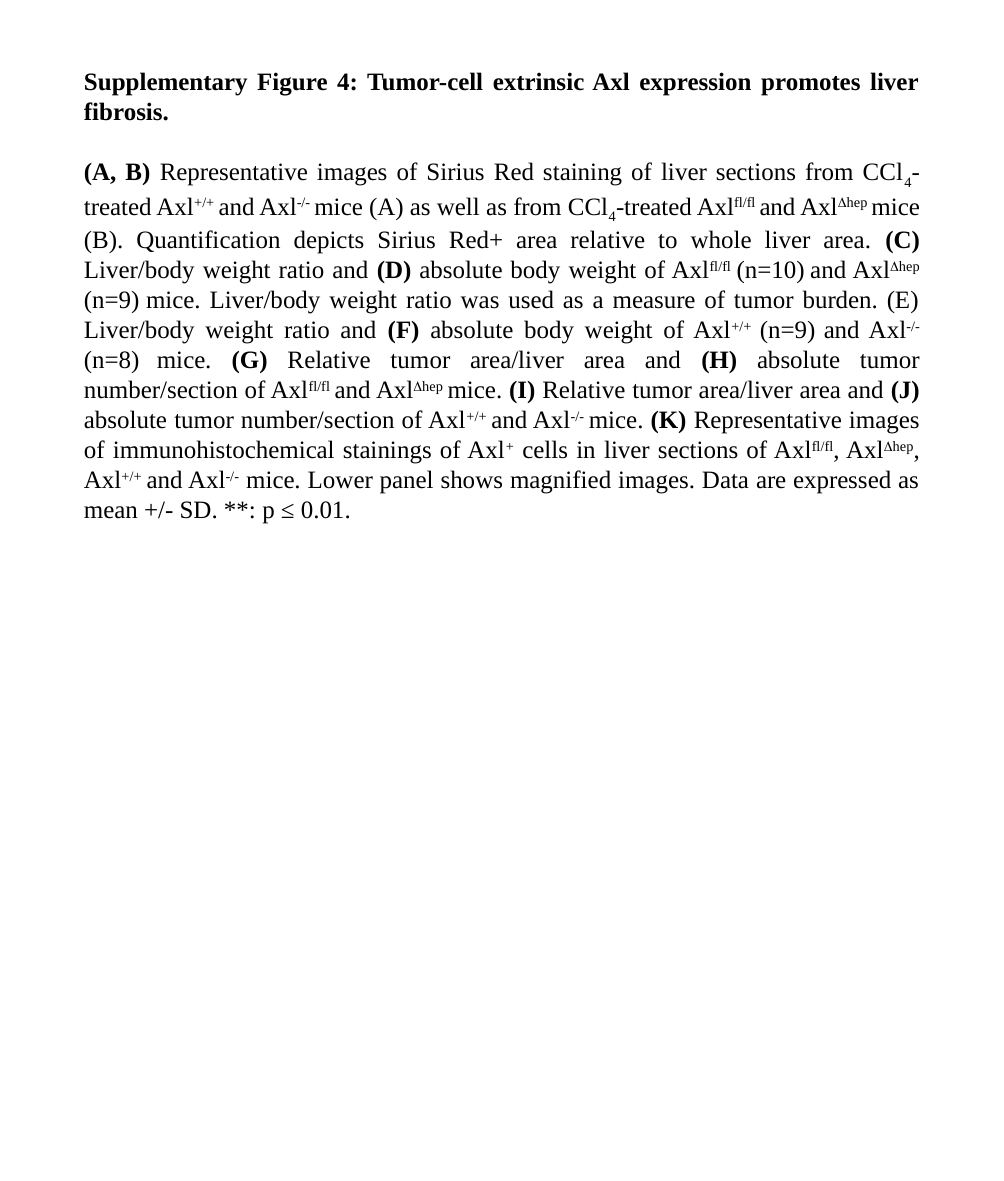

Supplementary Figure 4: Tumor-cell extrinsic Axl expression promotes liver fibrosis.
(A, B) Representative images of Sirius Red staining of liver sections from CCl4-treated Axl+/+ and Axl-/- mice (A) as well as from CCl4-treated Axlfl/fl and AxlDhep mice (B). Quantification depicts Sirius Red+ area relative to whole liver area. (C) Liver/body weight ratio and (D) absolute body weight of Axlfl/fl (n=10) and AxlDhep (n=9) mice. Liver/body weight ratio was used as a measure of tumor burden. (E) Liver/body weight ratio and (F) absolute body weight of Axl+/+ (n=9) and Axl-/- (n=8) mice. (G) Relative tumor area/liver area and (H) absolute tumor number/section of Axlfl/fl and AxlDhep mice. (I) Relative tumor area/liver area and (J) absolute tumor number/section of Axl+/+ and Axl-/- mice. (K) Representative images of immunohistochemical stainings of Axl+ cells in liver sections of Axlfl/fl, AxlDhep, Axl+/+ and Axl-/- mice. Lower panel shows magnified images. Data are expressed as mean +/- SD. **: p ≤ 0.01.

## Slide 7
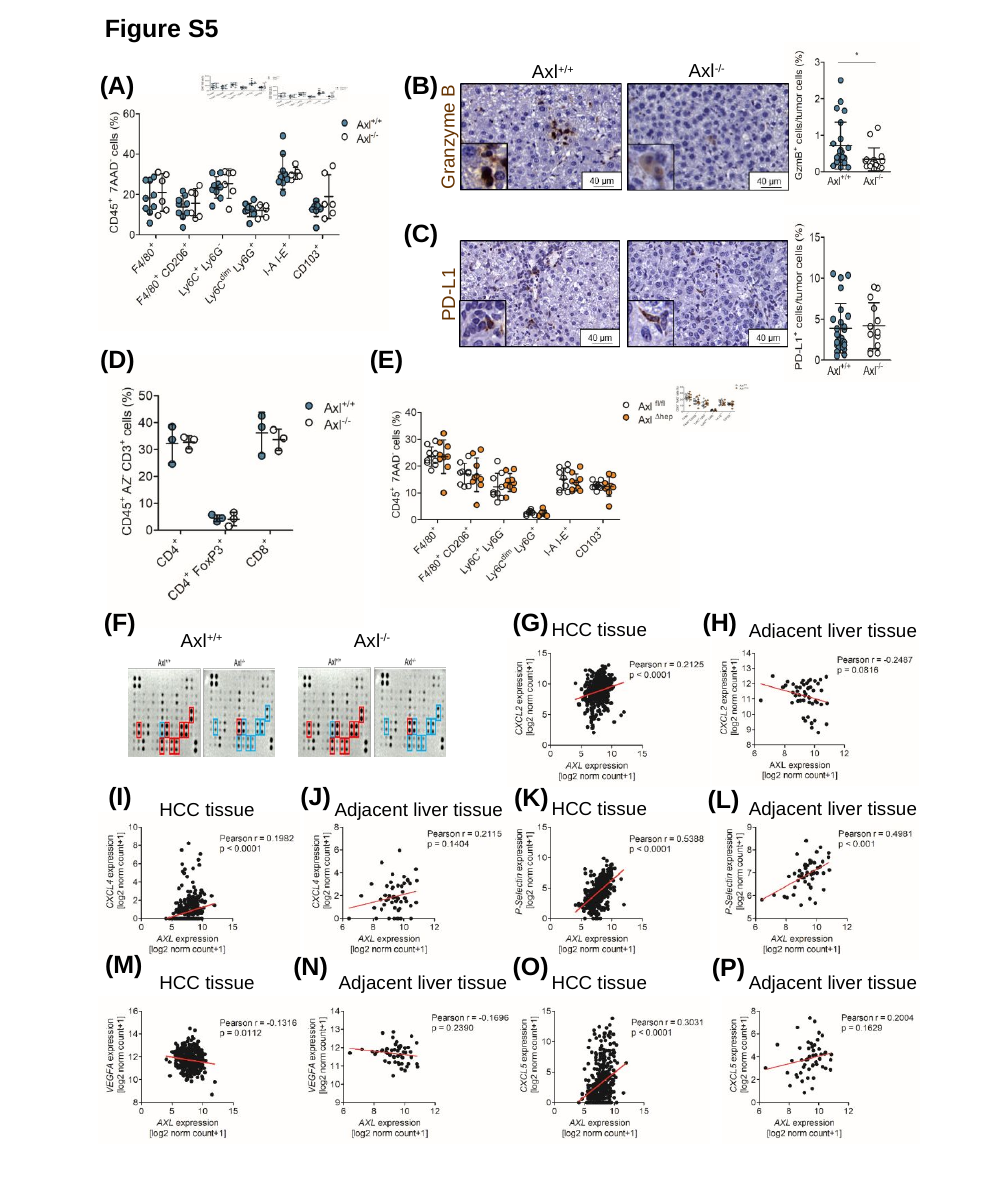

Figure S5
Axl-/-
Axl+/+
(A)
(B)
Granzyme B
(C)
PD-L1
(D)
(E)
(F)
(G)
(H)
HCC tissue
Adjacent liver tissue
Axl+/+
Axl-/-
(I)
(J)
(K)
(L)
HCC tissue
Adjacent liver tissue
HCC tissue
Adjacent liver tissue
(M)
(N)
(O)
(P)
HCC tissue
Adjacent liver tissue
HCC tissue
Adjacent liver tissue

## Slide 8
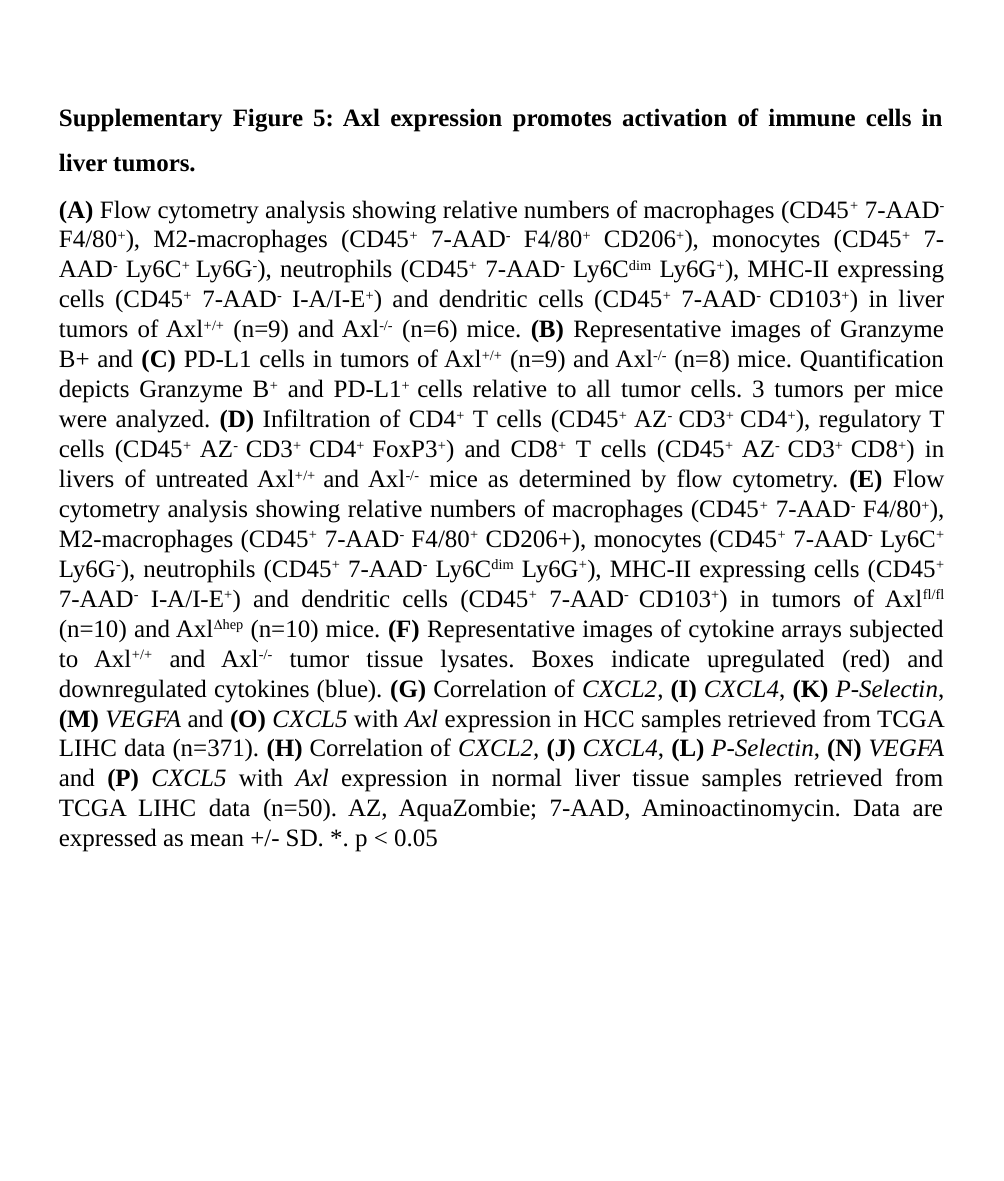

Supplementary Figure 5: Axl expression promotes activation of immune cells in liver tumors.
(A) Flow cytometry analysis showing relative numbers of macrophages (CD45+ 7-AAD- F4/80+), M2-macrophages (CD45+ 7-AAD- F4/80+ CD206+), monocytes (CD45+ 7-AAD- Ly6C+ Ly6G-), neutrophils (CD45+ 7-AAD- Ly6Cdim Ly6G+), MHC-II expressing cells (CD45+ 7-AAD- I-A/I-E+) and dendritic cells (CD45+ 7-AAD- CD103+) in liver tumors of Axl+/+ (n=9) and Axl-/- (n=6) mice. (B) Representative images of Granzyme B+ and (C) PD-L1 cells in tumors of Axl+/+ (n=9) and Axl-/- (n=8) mice. Quantification depicts Granzyme B+ and PD-L1+ cells relative to all tumor cells. 3 tumors per mice were analyzed. (D) Infiltration of CD4+ T cells (CD45+ AZ- CD3+ CD4+), regulatory T cells (CD45+ AZ- CD3+ CD4+ FoxP3+) and CD8+ T cells (CD45+ AZ- CD3+ CD8+) in livers of untreated Axl+/+ and Axl-/- mice as determined by flow cytometry. (E) Flow cytometry analysis showing relative numbers of macrophages (CD45+ 7-AAD- F4/80+), M2-macrophages (CD45+ 7-AAD- F4/80+ CD206+), monocytes (CD45+ 7-AAD- Ly6C+ Ly6G-), neutrophils (CD45+ 7-AAD- Ly6Cdim Ly6G+), MHC-II expressing cells (CD45+ 7-AAD- I-A/I-E+) and dendritic cells (CD45+ 7-AAD- CD103+) in tumors of Axlfl/fl (n=10) and AxlDhep (n=10) mice. (F) Representative images of cytokine arrays subjected to Axl+/+ and Axl-/- tumor tissue lysates. Boxes indicate upregulated (red) and downregulated cytokines (blue). (G) Correlation of CXCL2, (I) CXCL4, (K) P-Selectin, (M) VEGFA and (O) CXCL5 with Axl expression in HCC samples retrieved from TCGA LIHC data (n=371). (H) Correlation of CXCL2, (J) CXCL4, (L) P-Selectin, (N) VEGFA and (P) CXCL5 with Axl expression in normal liver tissue samples retrieved from TCGA LIHC data (n=50). AZ, AquaZombie; 7-AAD, Aminoactinomycin. Data are expressed as mean +/- SD. *. p < 0.05

## Slide 9
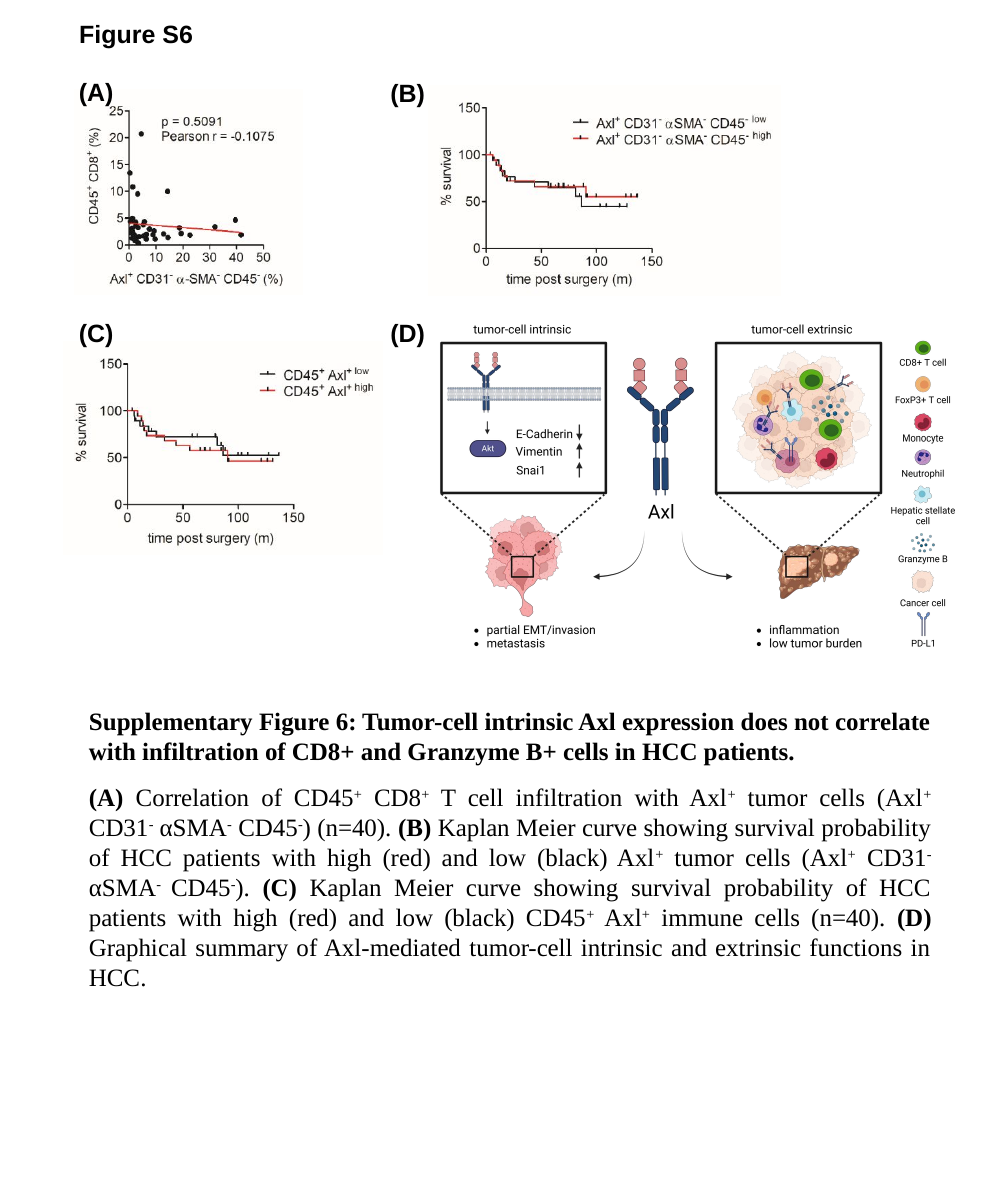

Figure S6
(A)
(B)
(C)
(D)
Supplementary Figure 6: Tumor-cell intrinsic Axl expression does not correlate with infiltration of CD8+ and Granzyme B+ cells in HCC patients.
(A) Correlation of CD45+ CD8+ T cell infiltration with Axl+ tumor cells (Axl+ CD31- αSMA- CD45-) (n=40). (B) Kaplan Meier curve showing survival probability of HCC patients with high (red) and low (black) Axl+ tumor cells (Axl+ CD31- αSMA- CD45-). (C) Kaplan Meier curve showing survival probability of HCC patients with high (red) and low (black) CD45+ Axl+ immune cells (n=40). (D) Graphical summary of Axl-mediated tumor-cell intrinsic and extrinsic functions in HCC.
